# PD-1 Expression and Cytokine Secretion Profiles of *Mycobacterium tuberculosis*-Specific CD4+ T-Cell Subsets; Potential Correlates of Containment in HIV-TB Co-Infection

**DOI:** 10.1371/journal.pone.0146905

**Published:** 2016-01-12

**Authors:** Katrina M. Pollock, Damien J. Montamat-Sicotte, Lisa Grass, Graham S. Cooke, Moses S. Kapembwa, Onn M. Kon, Robert D. Sampson, Graham P. Taylor, Ajit Lalvani

**Affiliations:** 1 Tuberculosis Research Centre, Respiratory Infections Section, National Heart and Lung Institute, Imperial College London, London, United Kingdom; 2 Section of Virology, Department of Medicine, Imperial College London, London, United Kingdom; 3 Department of GU and HIV Medicine, The North West London Hospitals NHS Trust, London, United Kingdom; 4 Tuberculosis Service, St Mary’s Hospital, Imperial College Healthcare Trust, London, United Kingdom; 5 Centre for Respiratory Infection, Flow Cytometry Facility, National Heart and Lung Institute, Imperial College London, London, United Kingdom; IPBS, FRANCE

## Abstract

HIV co-infection is an important risk factor for tuberculosis (TB) providing a powerful model in which to dissect out defective, protective and dysfunctional *Mycobacterium tuberculosis* (MTB)-specific immune responses. To identify the changes induced by HIV co-infection we compared MTB-specific CD4+ responses in subjects with active TB and latent TB infection (LTBI), with and without HIV co-infection. CD4+ T-cell subsets producing interferon-gamma (IFN-γ), interleukin-2 (IL-2) and tumour necrosis factor-alpha (TNF-α) and expressing CD279 (PD-1) were measured using polychromatic flow-cytometry. HIV-TB co-infection was consistently and independently associated with a reduced frequency of CD4+ IFN-γ and IL-2-dual secreting T-cells and the proportion correlated inversely with HIV viral load (VL). The impact of HIV co-infection on this key MTB-specific T-cell subset identifies them as a potential correlate of mycobacterial immune containment. The percentage of MTB-specific IFN-γ-secreting T-cell subsets that expressed PD-1 was increased in active TB with HIV co-infection and correlated with VL. This identifies a novel correlate of dysregulated immunity to MTB, which may in part explain the paucity of inflammatory response in the face of mycobacterial dissemination that characterizes active TB with HIV co-infection.

## Introduction

HIV co-infection is the greatest risk factor for progression of latent tuberculosis infection (LTBI) to active tuberculosis (TB) [1] and is associated with increased risk of *de novo* infection and re-infection [2]. Data from SIV or HIV-TB co-infection indicate an increase in the risk of disease reactivation prior to the onset of severe immunosuppression [3, 4]. This risk is not eliminated with HIV therapy remaining at least twice that of the general population [5]. HIV co-infection impacts the clinical phenotype of TB resulting in reduced cavitation, more severe disease, increased bacillary dissemination and poorer outcomes in terms of morbidity and mortality.

This early, specific and lasting effect on MTB-specific immunity makes HIV and LTBI co-infection a good model in which to dissect out defective, protective and dysfunctional MTB-specific immune responses. LTBI may be viewed as the successful containment of infection by effective anti-MTB immunity. Conversely, at the opposite end of the spectrum, active TB in the setting of HIV co-infection represents a manifest failure of MTB resistance control characterised by bacillary dissemination, high bacterial load and higher mortality. Cellular immune responses that specifically characterise this state, including cellular senescence [6] or immune exhaustion phenotypes, may provide insight into cellular mechanisms of failed MTB-specific immunity. MTB-specific cell subsets that are preferentially impaired or depleted by HIV infection are therefore candidates for correlation of immune containment and protective immunity.

Programmed death-1 (PD-1/CD279) is expressed by monocytes, CD4, CD8, NKT and B cells and binds to its ligands PDL-1 and 2 inhibiting proliferation and cytokine production [7]. Expression on total CD4+ and CD8+ T-cells correlates with viral load (VL) and on HIV-specific CD8+ T-cells mediates immune dysfunction. Blockade of the PD-1 axis on HIV-specific CD4+ and CD8+ cells can reverse this phenotype [7–11]. The PD-1 knock out mouse demonstrated a highly inflammatory response to MTB infection [12]. In humans with active TB, PD-1 expression correlated with IFN-γ production and prevention of ligand binding enhanced cellular immune function [13]. The effect of HIV and MTB co-infection in active and LTBI on the PD-1 axis in MTB-specific T-cells has not hitherto been investigated.

We hypothesized that in the setting of MTB infection, HIV co-infection would be associated with a reduced frequency of particular CD4+ MTB-specific T-cell cytokine secreting subsets and that this might serve as a correlate of MTB infection containment. Secondly, we hypothesised that HIV-TB co-infection would be associated with increased expression of PD-1 indicating a possible cellular correlate for the deficit in MTB-specific immunity. We studied four key clinical patient subgroups to delineate the impact of HIV in both stages of MTB infection. We used multi-parameter flow cytometry to measure three canonical cytokines and PD-1 expression in patients with active TB, TB and HIV co-infection, LTBI and HIV co-infection and LTBI as well as markers of T-cell exhaustion. This enabled simultaneous definition of functional and phenotypic MTB-specific T-cell profiles at the single-cell level.

## Materials and Methods

### Participant selection

Enrolment was from three London centres, UK during 2008–2011. The samples and clinical data used in this study were obtained from the TB Immunology Group Research Tissue Bank, which was approved by the St Mary’s Research Ethics Committee (07/H0712/85). The Committee is constituted in accordance with the Governance Arrangements for Research Ethics Committees (July 2001) and complies fully with the Standard Operating Procedures for Research Ethics Committees in the UK. All samples were donated to the tissue bank after the subjects had given written, informed consent and the research was approved by the Tissue Bank Steering Committee in accordance with the approved procedures for Tissue Banks in the UK. Eligibility criteria included current investigation for active TB or LTBI or the presence of risk factors for LTBI. All individuals underwent immune testing for MTB infection using a *Mycobacterium tuberculosis*-specific blood test (IFN-γ MTB ELISpot, as described below). Active TB cases were all microbiologically confirmed. LTBI cases excluded those with symptomatic, microbiological or radiological evidence of active TB. HIV infection was confirmed according to national standards and HIV VL and CD4 T-lymphocyte counts were assayed in the local Clinical Pathology Association accredited diagnostic laboratories at the time of study recruitment. Participants were selected for flow cytometry analysis if they met strict case definition criteria for LTBI, active TB and/or HIV co-infection.

### Definition of LTBI

A positive IFN-y response to ESAT-6 and CFP-10 and/or positive response to novel validated MTB-specific peptides including MTB-specific peptides from Rv3873 [14, 15] and Rv3615c[16]. Individuals with HIV co-infection were enrolled if they had risk factors for LTBI and screened for the presence of LTBI [17]. All individuals with LTBI had a positive interferon gamma release assay (IGRA) for MTB infection and/or tuberculin skin test.

### Antigens

PPD was obtained from Serum Statens Institute and re-suspended in RPMI at 16.7μg/ml final concentration. Pools of MTB-specific 15-mer overlapping peptides covering each of ESAT-6, CFP-10, and Rv3615c were re-suspended in a single mixture (10μg/ml per peptide final concentration).

### IFN-γ MTB ELISpot

Fresh or frozen peripheral blood mononuclear cells (PBMCs), 2.5 x 10^5^ per well, were left unstimulated or stimulated overnight (37°C 5% CO_2_ for 16 to 20 hours) with PHA, purified protein derivative (PPD) and pools of MTB-specific 15-mer overlapping peptides covering each of ESAT-6, CFP-10, EspC, TB7.7, Rv3879c, Rv3873 and Rv3878 in an IFN-γ capture ELISpot assay as previously described [18]. Spot forming cells were counted using an AID ELISpot reader. A positive response was at least 5 spot-forming cells more than the negative control well and at least twice the negative control well. This method of diagnosing MTB infection in the context of clinical data has been widely validated [19–22].

### Intracellular cytokine staining

The cells were prepared as previously described [23], thawed PBMCs (3–5 x 10^6^ per well) were cultured for 16 hours (37°C 5% CO_2_) in 10% human serum in Roswell Park Memorial Institute (RPMI) medium. Cells were stimulated with PMA-Ionomycin, (5ng/ml PMA and 500ng/ml ionomycin final concentration), PPD or MTB-specific peptides or left unstimulated. After 2 hours Monensin (2 μM final concentration) was added. Cells were stained with a dead cell discriminator (LIVE/DEAD^®^ Fixable Dead Cell Stain Kits, aqua, Invitrogen) (30 minutes, 4°C) washed and placed in FC block buffer (10% human serum in filtered FACS solution: 0.5% bovine serum albumin and 2mM EDTA in PBS) (20 minutes at 4°C). Cells were stained with titrated fluorochrome-conjugated antibodies against CD3-APC-Alexa Fluor^®^750, CD4-Qdot^®^605 and CD45RA-Qdot^®^655 (Invitrogen), CD8-APC, CCR7-PE-Cy^™^7 and CD127-FITC (BD Biosciences) and PD-1-PerCP/Cy5.5 (Biolegend) then fixed and permeabilised with BD Cytofix/Cytoperm^™^ Fixation/Permeabilization kit (BD Biosciences) (20 minutes, 4°C). Cells were washed twice with Perm/Wash solution (BD Biosciences) were stained with optimised fluorochrome-conjugated antibodies, IFN-γ-V450, IL-2-PE and TNF-α-AlexaFluor 700 (BD Biosciences) (30 minutes 4°C). Where possible, 1x10^6^ events were acquired using a BD LSR-II flow cytometer and compensation parameters set with Anti-Rat and Anti-Mouse Ig compensation beads (BD Biosciences). Fluorescence minus one (FMO) controls were included each time. Data on CD8, CCR7, CD45RA and CD127 are not included in this report, having been published separately but are included in the methods for accuracy [23, 24].

### Data analysis

FlowJo©TreeStar, Inc. v9.2 was used to gate on live cells, singlets and lymphocytes and CD3+CD4+ subsets. FMO controls were used to define positive gates for IFN-γ, IL-2 and TNF-α responses and PD-1 expression. Non-overlapping subsets of cytokine response were created using Boolean analysis. Frequencies were normalised to the unstimulated control. Responders had a response ≥2 times the background and >0.001% of CD3+CD4+ T-cells and were analysed for PD-1 expression. Those without a positive response were excluded from phenotypic analysis. Co-stimulatory molecules were not included in the assay to avoid signals not necessarily present in vivo. Using these stringent cut-off criteria, antigen-specific rare events were assumed to be real events.

### Statistical analysis

This was conducted using IBM SPSS Statistics version 20 and Prism version 5. The Mann-Whitney U test was used for two sample comparisons and Kruskal-Wallis test with Dunn’s post-test comparison for multiple comparisons. Spearman’s Rank coefficient for tests of correlation of non-parametric data was used. CD4 count and HIV VL were analysed as continuous variables. A VL of 10 was designated where viral suppression was achieved.

## Results

### Participant characteristics

All individuals had a positive response to ESAT-6 or CFP-10 in the MTB-specific IFN-γ ELISpot or T.Spot*TB* except one who had a TST >15mm and a positive response to Rv3615c. Routine HIV-screening is currently not undertaken in the UK for LTBI. All five patients where an HIV test had not been clinically indicated had normal CD4/CD8 ratios. The median (IQR) CD4 count (cells/μl) of those with TB/HIV and LTBI/HIV was 180 (136–200) and 455 (356–530) and the median (IQR) VL (RNA copies/ml) was 28,958 (66–281,671) and 10 (10–12,691) (Table 1). All individuals with TB or LTBI had received <14 days treatment for MTB except one. 8 individuals with HIV infection were taking HAART at the time of blood sampling; two (28.6%) TB/HIV subjects and six (60.0%) LTBI/HIV subjects, of whom 7 had suppressed viral loads and one had a viral load of 66.

**Table 1:** Participant characteristics.

	TB/HIV		TB		LTBI/HIV		LTBI		Total	
	7	(%/IQR)	6	(%/IQR)	10	(%/IQR)	11	(%/IQR)	34	(%/IQR)
Age	43	(40,54)	35	(26,57)	37	(24,40)	34	(30,36)	36	(31,41)
Male	4	(57.1)	3	(50.0)	6	(60.0)	4	(36.4)	17	(50.0)
Female	3	(42.9)	3	(50.0)	4	(40.0)	7	(63.6)	17	(50.0)
Black African	5	(71.4)	1	(16.7)	8	(80.0)	6	(54.5)	20	(58.8)
Asian	1	(14.3)	4	(66.7)	0	(0.0)	3	(27.3)	8	(23.5)
Caucasian	1	(14.3)	1	(16.7)	2	(20.0)	2	(18.2)	6	(17.6)
HIV positive	7	(100.0)	0	(0.0)	10	(100.0)	0	(0.0)	17	(50.0)
HIV negative or untested^a^	0	(0.0)	6	(100.0)	0	(0.0)	11	(100.0)	17	(50.0)
CD4 cells/μl	180	(136;200)	NA	NA	455	(356;530)	NA	NA	NA	NA
HIV viral load	28,958	(66;281,671)	NA	NA	10	(10;12,691)	NA	NA	NA	NA
HIV treated	2	(28.6)	NA	NA	6	(60.0)	NA	NA	NA	NA
HIV untreated	5	(71.4)	NA	NA	4	(40.0)	NA	NA	NA	NA
BCG vaccinated	5	(71.4)	3	(50.0)	9	(90.0)	8	(72.7)	25	(73.5)
BCG un vaccinated	1	(14.3)	2	(33.3)	0	(0.0)	2	(18.2)	5	(14.7)
not known	1	(14.3)	1	(16.7)	1	(10.0)	1	(9.1)	4	(11.8)
IGRA or tuberculin skin test positive	7	(100.0)	6	(100.0)	10	(100.0)	11	(100.0)	34	(100.0)
MTB infection confirmed	7	(100.0)	6	(100.0)	NA	NA	NA	NA	NA	NA

^a^ 5 individuals with LTBI did not undergo testing for HIV infection but had normal CD4/8 ratios

IGRA interferon gamma release assay

IQR interquartile range

### MTB-specific CD4+ T-cells secreting IFN-γ and IL-2 correlated inversely with HIV viral load

**Fig 1:**
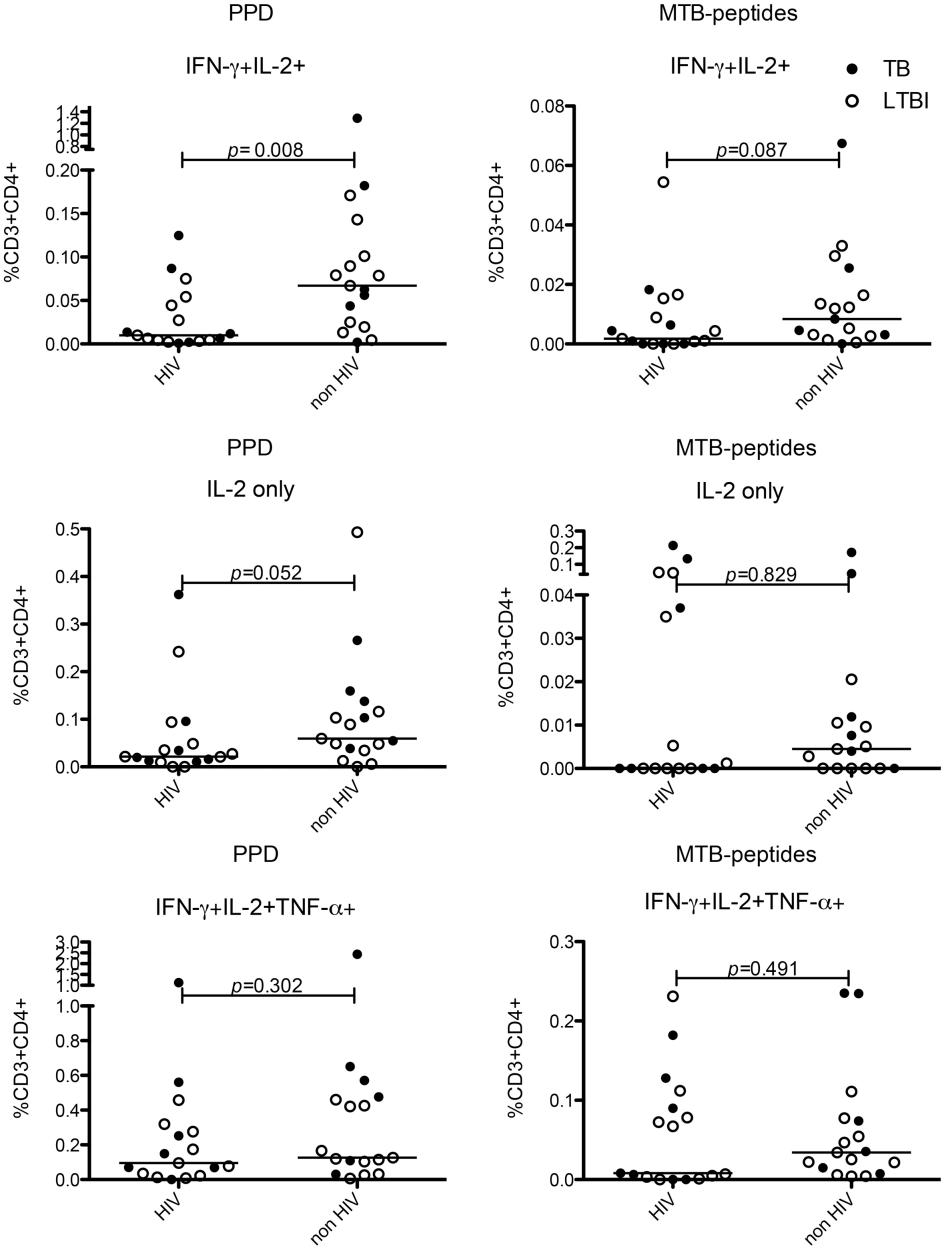
HIV co-infection negatively impacts MTB-specific CD4+ functional cell subsets secreting IFN-γ and IL-2. Graphs show frequency and median of CD4+ cell functional subsets secreting IFN-γ and IL-2 (top row) and IL-2 only (middle row) and IFN-γ, IL-2 and TNF-α (bottom row) in response to overnight stimulation with PPD (left column) and MTB-peptides (right column). p values on graphs show Mann-Whitney U tests for HIV-infected vs. HIV-uninfected (n = 34). Closed circles represent those with TB, and open circles those with LTBI with or without HIV co-infection.

We used Boolean gating to create 7 non-overlapping T-cell subsets secreting single or combinations of 3 cytokines (IFN-γ, IL-2 and TNF-α) (S1 Fig). The frequency of these subsets was compared to examine the impact of both HIV infection and active TB infection compared with LTBI on MTB-specific T-cell responses. CD4+ MTB-specific T-cells secreting both IFN-γ and IL-2 were reduced in frequency in individuals with HIV-TB co-infection (whether active or latent) unlike other IL-2 secreting subsets (Fig 1). There was no difference in absolute frequency of any CD4+ subset responding to PPD or MTB-peptides in those on HAART compared with those not on HAART, however there was a trend towards a lower proportion of IFN-γ and IL-2 dual secreting cells in those not taking HAART (S2 Fig). There was no difference in the frequency of MTB-specific CD4+ T-cells secreting IFN-γ and IL-2 in response to PPD or MTB-peptides in individuals with active TB/HIV compared with LTBI/HIV or in those with active TB compared with LTBI without HIV co-infection (data not shown). However the proportion of the total response to PPD or MTB peptides contributed by CD4+ T-cells secreting IFN-γ and IL-2 together was lowest in active TB/HIV compared with LTBI without HIV co-infection (S3 Fig).

**Fig 2:**
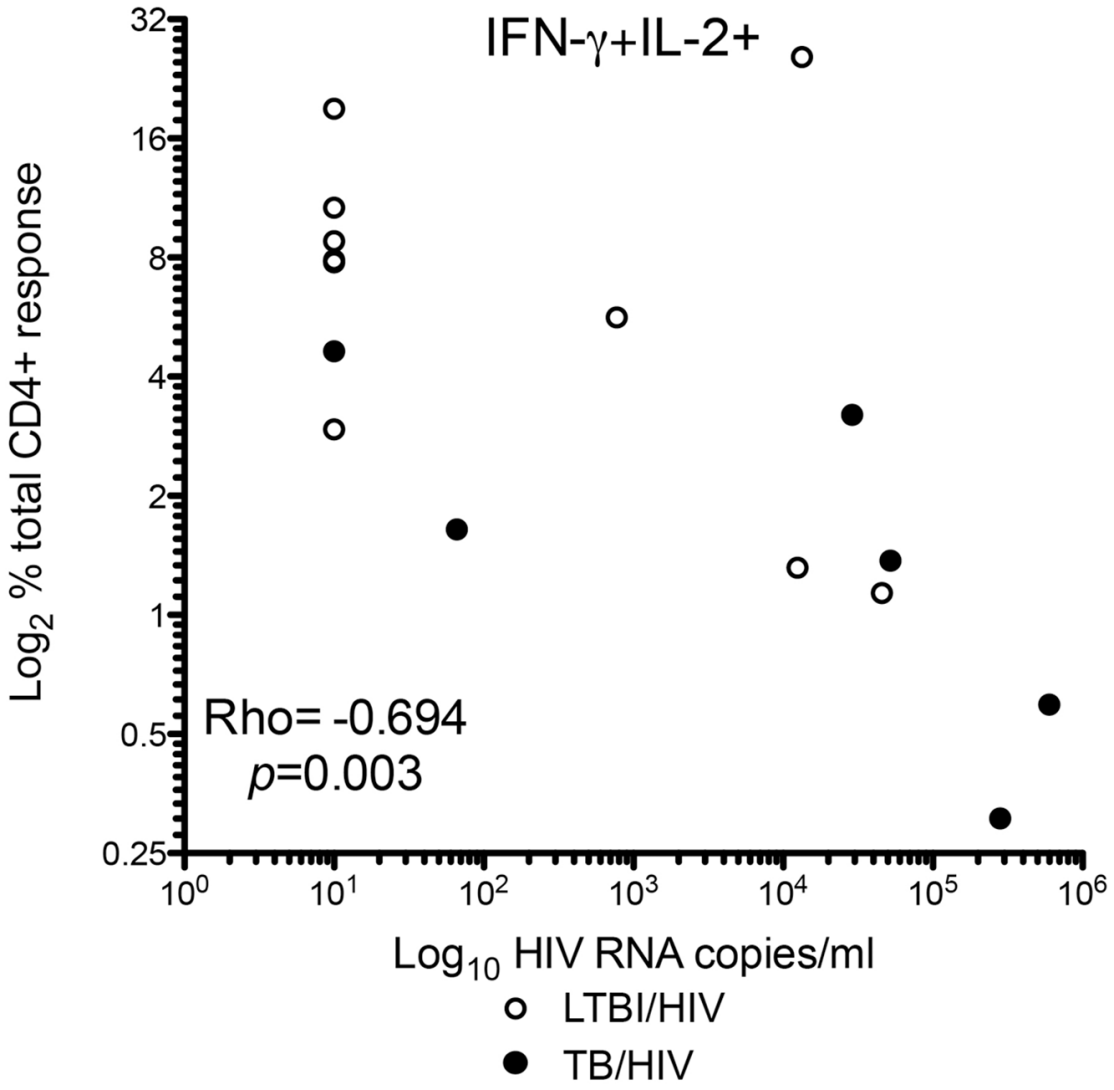
HIV VL is negatively correlated with the proportion of CD4+ MTB-specific T-cells secreting IFN-γ and IL-2. Graph shows Spearman’s rank correlation of proportion of PPD-specific CD4+ IFN-γ and IL-2-dual-secreting cells with HIV VL. Participants with both active TB/HIV (closed circles) and LTBI/HIV (open circles) and a positive response to PPD are shown (n = 16).

There was an inverse relationship between the proportion of MTB-specific CD4+ IFN-γ and IL-2-dual-secreting cells contributing to the total CD4+ T-cell cytokine response and HIV VL (Rho = -0.694, p = 0.003) (Fig 2). Neither HIV VL nor CD4 count correlated with the proportion of MTB-specific CD4+ IL-2-only-secreting T-cells. There was a weak association of CD4 count with the proportion of MTB-specific tri-functional cells (Rho = 0.525, p = 0.037). The proportion of MTB-specific CD4+ T-cell functional effectors secreting IFN-γ, TNF-α or both was inversely correlated with CD4 count (IFN-γ only Rho = -0.518, p = 0.040 and dual Rho = -0.633 p = 0.009) and correlated with HIV VL (TNF-α only Rho = 0.664, p = 0.005).

### PD-1 expression on PPD-specific T-cells was related to HIV and active TB co-infection and correlated with HIV viral load

**Fig 3:**
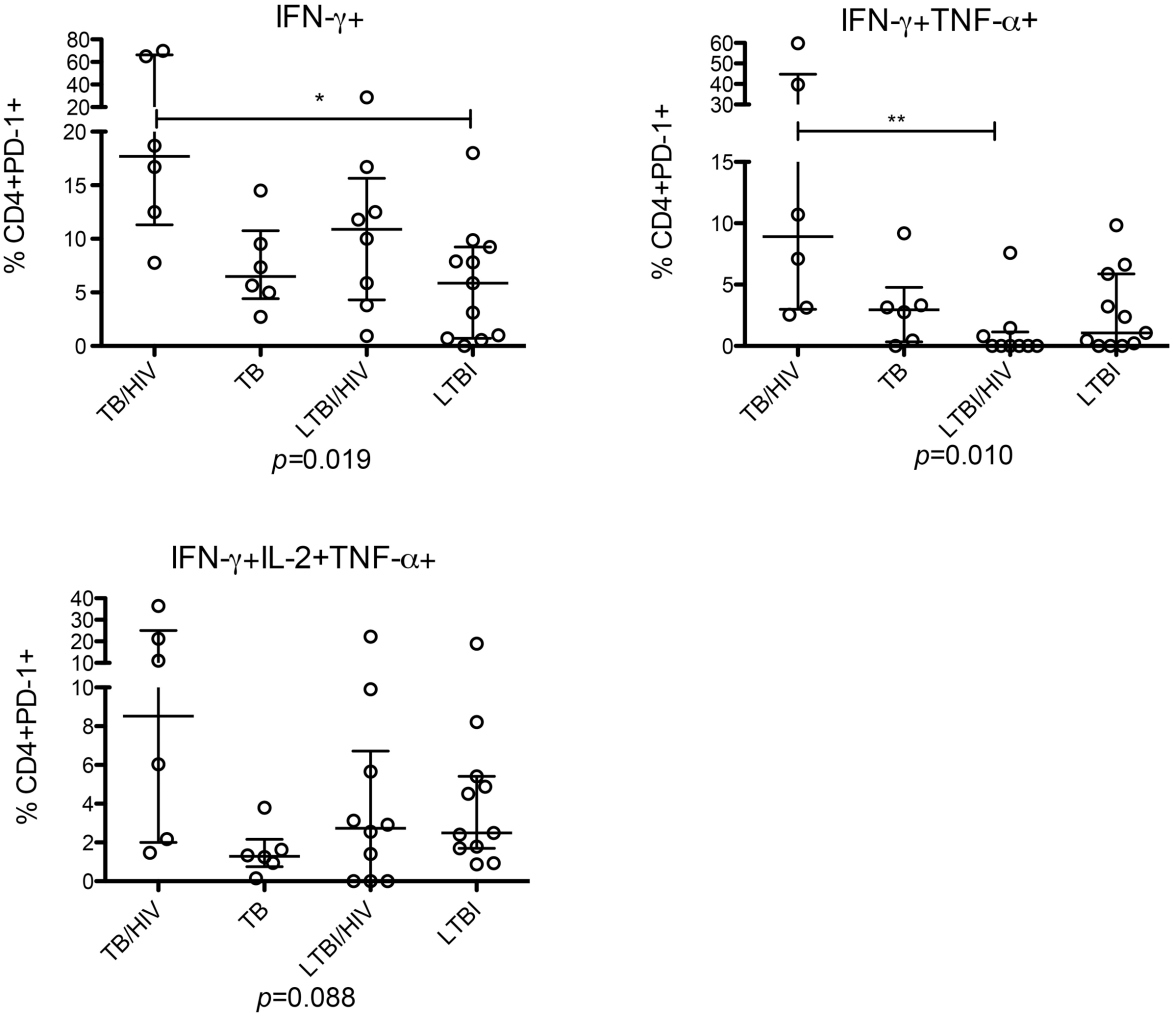
PD-1 expression by MTB-specific T-cells secreting IFN-γ is increased in active TB /HIV co-infection. Graphs show percentage, median and IQR of PD-1 expression on CD4+ subsets secreting IFN-γ-only (top left), IFN- γ and TNF-α (top right) and IFN-γ, IL-2 and TNF-α (bottom left) following overnight stimulation with PPD. Individuals without a positive response were excluded. p values show Kruskal-Wallis test with Dunn’s post-test comparison.

We next investigated the expression of PD-1 (in those with a positive response) on MTB- specific T-cells (S4 Fig). Active TB with HIV co-infection was associated with a higher percentage of MTB-specific CD4+ T-cells secreting IFN-γ alone expressing PD-1 compared to all other groups, p = 0.019 (Fig 3). Similarly the greatest frequency of PD-1 expression in MTB-specific cells secreting IFN-γ and TNF-α was seen in patients with active TB /HIV co-infection (p = 0.010) with a similar trend in tri-functional cells (Fig 3). The highest rates of PD-1 expression were therefore seen in patients with active TB / HIV co-infection.

**Fig 4:**
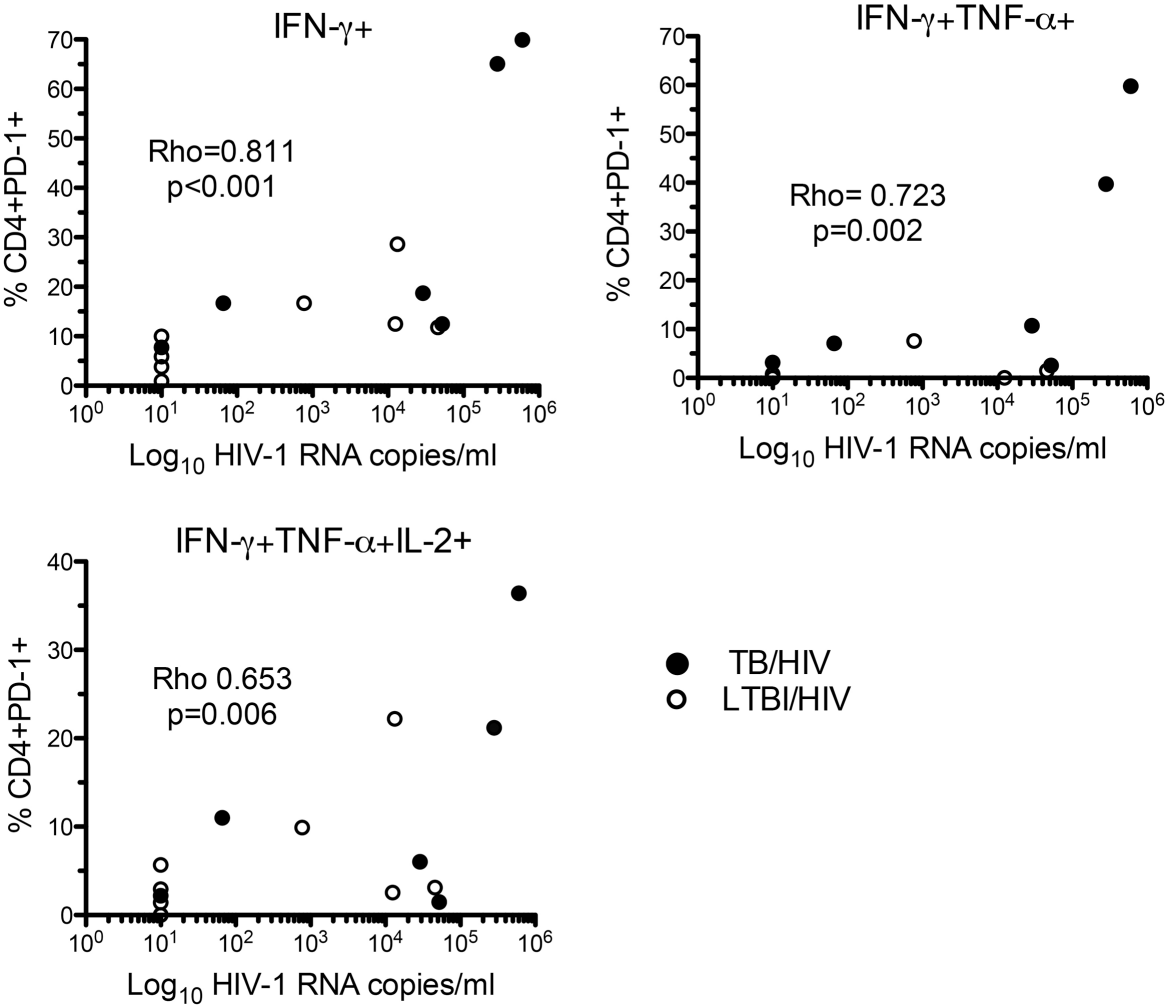
Association of PD-1 expression on CD4+ MTB-specific T-cells secreting IFN-γ with HIV VL. Graphs show Spearman’s rank correlation of HIV VL with the percentage of cells expressing PD-1, for PPD-specific CD4+ cells secreting IFN-γ-only (top left), IFN- γ and TNF-α (top right) and IFN-γ, TNF-α and IL-2 (bottom left). Those without a positive response in the relevant functional subset were excluded. Those with TB/HIV (closed circles) and LTBI/HIV (open circles) are shown.

HIV VL correlated with the percentage of MTB-specific CD4+ IFN-γ-only-secreting cells that expressed PD-1, independently of the frequency of CD4+ IFN-γ-only secreting cells (Rho = 0.811, p<0.001) (Fig 4). The percentage of MTB-specific CD4+ IFN-γ and TNF-α-dual (Rho = 0.723, p = 0.002) and CD4+ tri-functional- (Rho = 0.653, p = 0.006) cells responding to PPD (Fig 4) and CD4+IFN-γ and TNF-α-dual-secreting cells responding to MTB peptides (Rho = 0.628 p<0.001) (data not shown) expressing PD-1 correlated with HIV VL. There was an inverse correlation of the percentage of cells expressing PD-1 with CD4 count for PPD-responsive CD4+ IFN-γ and TNF-α-dual-secreting cells (Rho = -0.618 p = 0.014), MTB-peptides-responsive IFN-γ and TNF-α-dual (Rho = -0.802 p = 0.001) and IFN-γ-only-secreting cells (Rho = -0.757 p = 0.021). These relationships were not observed in cell subsets not secreting IFN-γ and overall the greatest PD-1 expression was seen in those with active TB / HIV co-infection. A lower proportion of CD4+ MTB-specific cells, secreting IFN-γ alone, IFN-γ plus TNF-α and all three cytokines in response to PPD, expressed PD-1, in those on HAART compared with those not on HAART (p = 0.010, p = 0.031 and p = 0.031 respectively).

## Discussion

### CD4+ MTB-specific cells secreting IFN-γ and IL-2 may serve as a correlate of containment in HIV-TB co-infection

Our data indicate potential HIV-induced correlates underlying the loss of containment of MTB and the immune dysfunction in active TB contributing to the early and lasting effects of HIV co-infection and increased TB-related morbidity and mortality [4, 25, 26]. We previously showed that HIV infection was associated with a reduced frequency of MTB-specific IFN-γ and IL-2 dual-secreting CD4+ cells in patients with LTBI, most of whom had normal or near normal CD4 counts due to immune restoration with HAART [24]. Here we see a greater impact on this subset in patients that have not yet started HAART and furthermore an inverse correlation with HIV VL. We found very little evidence of relationships between other MTB-specific CD4+ T-cell subsets and HIV infection pinpointing IFN-γ and IL-2-dual secreting cells as uniquely impacted by HIV infection. This may reflect an increasing inability to contain MTB in individuals with uncontrolled HIV replication, although we were unable to prove causality in this cross sectional study. Our findings are consistent with the cumulative evidence for an association of antigen-specific CD4+ cells secreting IL-2 with or without IFN-γ in the containment or resolution of viral and TB infections [24, 27–33]. The association of HIV VL with reduced frequency of IL-2 secreting cells has been investigated but without an HIV-uninfected control group for comparison [32, 34]. The relationship with increasing VL is consistent with an effect mediated by on-going viral replication, such as specific killing but teasing out the mechanisms of this HIV-induced antigen-specific cell death is complex. MTB-specific cells may be lost early in HIV infection and IL-2-secreting cells may be more vulnerable to infection with HIV [35, 36].

### MTB-specific CD4+ functional effector responses and PD-1 expression may reflect mycobacterial burden

The associations of MTB-specific CD4+ cells secreting IFN-γ with or without TNF-α (CD4+ effector-like cells) with CD4 count and VL that we demonstrated likely reflects increasing mycobacterial burden in patients with HIV and TB as previously shown [23, 37]. Active TB with advanced HIV co-infection was characterised by a high frequency of the MTB-specific IFN-γ-only-secreting subset and the expression of PD-1 may be linked to IFN-γ secretion [13]. Increased expression of PD-1 on CD4+ MTB-specific IFN-γ-secreting T-cells was associated with HIV co-infection and correlated with VL. Given the role of PD-1 in immune regulation and exhaustion and its close correlation with total CD4+ T-cell activation and immunosuppression in HIV infection [8, 11, 38–40], these data suggest a novel correlate of dysregulated immunity that is a feature of active TB/HIV. Whilst these changes were also noted in those with LTBI and HIV co-infection the greatest effect was in those with active TB and HIV co-infection. Although our analysis of PD-1 expression on MTB-specific T-cells showed a correlate with HIV VL this was most marked in the patients where mycobacteria were likely to be disseminated. HIV VL may therefore be acting as a surrogate marker of mycobacterial burden driving PD-1 expression through T-cell receptor stimulation.

Studies in mice and humans indicate that in TB, PD-1 might function as a self-regulatory and tissue-protective off-switch [12, 13, 41–44]. Our findings on MTB-specific T-cells in HIV-TB co-infection might therefore underlie two observations that relate to the immune ‘exhaustion’ and tissue-protective functions of PD-1. The increased expression of PD-1 on IFN-γ-secreting CD4+ T-cells might contribute to reduced ability to control MTB infection and underpin the paucity of immunopathology as manifested by, for example, the lack of cavitatory disease resulting in the increased bacillary load and dissemination that characterize active TB/HIV.

### Role of tri-functional cells

The frequency of MTB-specific CD4+ tri-functional T-cells secreting IFN-γ, IL-2 and TNF-α was independent of both mycobacterial burden and HIV co-infection. Tri-functionality has been associated with active TB [45, 46] but data from the mouse lung suggests an alternative protective role [47] and polyfunctionality is a putative marker of protective immunity in vaccine trials [48]. We did not find sufficient evidence to suggest the tri-functional CD4+ T-cell subset correlates with protection although there was a weak association in those who were highly immunosuppressed. Some data have suggested that polyfunctional, less differentiated cells might be increased at the site of extra-pulmonary TB in those with HIV co-infection [49]. Our data indicated that in PBMCs, neither the presence of polyfunctionality nor the frequency of tri-functional cells was significantly affected by HIV co-infection or TB disease stage, which detracts from its putative role as a peripheral marker of protection [50] or active TB [46], however in our limited sample size the presence of weak associations cannot be excluded.

### Effect of HAART on MTB-specific CD4+ cell responses

The natural history of HIV and TB co-infection means that extricating the effects of HAART from the effects of mycobacterial burden is challenging and beyond the scope of this study. We were unable to control for the presence of HAART and although HIV VL may be taken as a surrogate marker for its effects, the immunological outcomes we observed cannot be disentangled from associated changes in mycobacterial burden.

### Limitations of this work

Comprehensive analysis of a small number of precisely defined subjects has enabled us to study MTB-specific T-cell responses in detail and to uncover the most significant relationships. Other possible immunological effects cannot be ruled-out on account of our small sample size. We are unable to infer causality based solely on the associations identified in this cross-sectional study. Nevertheless, taken together these findings may have significance for monitoring disease activity in MTB-infected individuals.

## Conclusions

Our detailed dissection of the effects of HIV-TB co-infection on the MTB-specific CD4+ T-cell immune response has provided insight into clinically important features and indicated potentially useful immune correlates for monitoring disease activity. Our data accords with mounting evidence that MTB-specific T-cells secreting IL-2 with or without IFN-γ have a protective role to play in immune containment and suggests that the dual-secreting cells may be of greatest significance in this capacity. Secondly we identified a correlate of exhausted and dysregulated antimycobacterial immunity in active TB with advanced HIV co-infection that may partially explain certain characteristic clinical features of this life-threatening condition. Our hypothesis is that the immunophenotype (cytokine secretion profile and expression of PD-1) of the MTB-specific CD4+ T-cell response reflects the manifest failure of MTB containment in HIV co-infection *i*.*e*. active TB. Clinical observations suggest that in the presence of HIV infection this failure is profound with widespread dissemination of MTB. This is indicated by both the loss of CD4+ IFN-γ and IL-2 secreting cells and the increased frequency of PD-1 expression, which we observed in TB and HIV co-infection. If large longitudinal studies can prospectively validate our findings, these correlates may help to pave the way for the development and evaluation of new interventions to augment immune containment in LTBI and reverse immune dysregulation in active TB.

## Supporting Information

S1 Fig Boolean gating of MTB-specific CD4+ functional cell subsets. PBMCs from donors were left unstimulated or stimulated with PPD or MTB-specific peptides for 16 hours with Monensin added after 2 hours. The cells were stained with a dead cell discriminator then fluorescence-conjugated antibodies against phenotypic markers before fixing, permeabilising and staining with antibodies targeting intracellular IFN-γ, IL-2 and TNF-α. Plots are representative of one individual with active TB/HIV and show PBMCs stimulated with PPD. Single, live, CD3+ lymphocytes were gated for CD4+ and CD8+ cells (top row). Gating was performed in two dimensions for quadrants containing IFN-γ, IL-2 and TNF-α positive and negative cells, (middle row). Three-dimensional Boolean gating was used to select cytokine subsets; an example of CD3+CD4+ IFN-γ-only-secreting cells is shown (bottom row) selecting only IFN-γ-only-secreting cells from quadrants in the middle row (Q1, Q5 and Q12). Multi-dimensional Boolean gating allows the user to exclusively select subsets secreting specific cytokines, which is represented 2-dimensionally here. For example selecting IFN-γ-only-secreting cells excludes all cells secreting IL-2 or TNF-α.
(EPS)

S2 Fig Frequency and proportion of CD4+ MTB-specific IFN-γ and IL-2 dual secreting cells by HIV treatment status. Graphs show frequency of CD4+ MTB-specific T-cells secreting IFN-γ and IL-2 (top) and proportion of CD4+ MTB-specific T-cells secreting IFN-γ and IL-2 (bottom) in those with HIV infection on therapy or not on therapy. Those with TB/HIV (closed circles) and LTBI/HIV (open circles) are shown.
(EPS)

S3 Fig Proportion of MTB-specific CD4+ T-cells secreting IFN-γ and IL-2 of the total response where there was a positive response. Upper panel shows response to PPD and lower panel to MTB-peptides. Graphs show results of Kruskal-Wallis test with Dunn’s post-test comparison.
(EPS)

S4 Fig Gating strategy for PD-1 expression by MTB-specific CD4+ T-cells. Plots are representative of one individual with active TB and HIV and show PBMCs stimulated with PPD. Live, CD3+ lymphocytes were gated for CD4+ and CD8+ cells, then for quadrants containing IFN-γ, IL-2 and TNF-α positive and negative cells, Boolean gating was used to select cytokine subsets in 3 dimensions to generate 7 cytokine subsets, an example of CD3+CD4+ IFN-γ-only secreting cells is shown and PD-1 expression was determined using PerCP-Cy5.5 fluorescence minus one as a gating control.
(EPS)
